# Molecular anatomy of the gut-brain axis revealed with transgenic technologies: implications in metabolic research

**DOI:** 10.3389/fnins.2013.00134

**Published:** 2013-07-31

**Authors:** Swalpa Udit, Laurent Gautron

**Affiliations:** Division of Hypothalamic Research, Department of Internal Medicine, University of Texas Southwestern Medical Center at DallasDallas, TX, USA

**Keywords:** vagus nerve, mouse models, autonomic nervous system, morphology, obesity

## Abstract

Neurons residing in the gut-brain axis remain understudied despite their important role in coordinating metabolic functions. This lack of knowledge is observed, in part, because labeling gut-brain axis neurons and their connections using conventional neuroanatomical methods is inherently challenging. This article summarizes genetic approaches that enable the labeling of distinct populations of gut-brain axis neurons in living laboratory rodents. In particular, we review the respective strengths and limitations of currently available genetic and viral approaches that permit the marking of gut-brain axis neurons without the need for antibodies or conventional neurotropic tracers. Finally, we discuss how these methodological advances are progressively transforming the study of the healthy and diseased gut-brain axis in the context of its role in chronic metabolic diseases, including diabetes and obesity.

“These nerves [to the bowels] are but small, because the parts serving for nutrition, needed none but little nerves, for the performance of the third duty of the nerves, which is in the discerning and knowing of what is troublesome to them […]. We have this benefit by this sense, that as soon as anything troubles and vellicates the bowels, we being admonished thereof may look for help in time”

–Ambroise Paré, circa 1579.

## Overview of the anatomical study of the gut-brain axis

**Figure 1:**
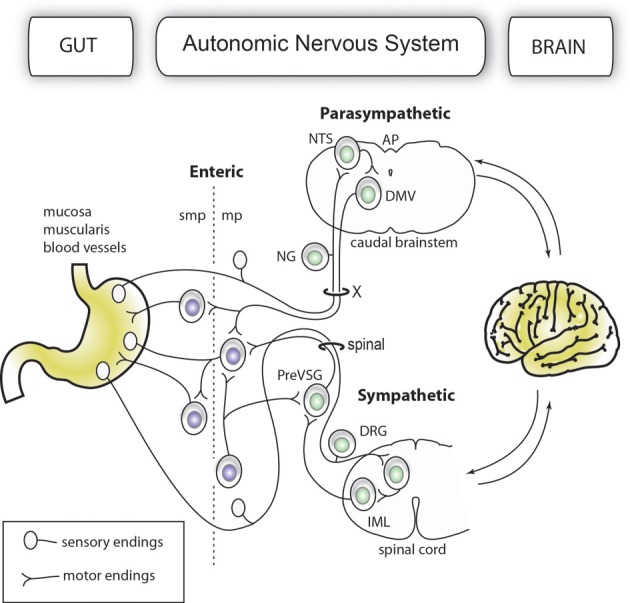
**Simplified organization of the gut-brain axis**. The main anatomical and functional subdivisions of a mammalian gut-brain axis are represented with neurons belonging to the enteric and extrinsic autonomic systems in different colors. Importantly, the enteric nervous system is entirely contained within the gastrointestinal wall. For purposes of simplification, all the nerve branches supplying the gut, pancreatic post-ganglionic neurons and the sacral parasympathetic system are not depicted in this figure.

The gut-brain axis comprises a network of autonomic neurons that connect the central nervous system (CNS)—specifically, the caudal brainstem and spinal cord—to the esophagus, gastrointestinal tract, liver, and pancreas (Loewy and Spyer, 1990; Janig, 1996; Powley, 2000; Gibbins et al., 2003; Furness, 2006). The axons of these neurons travel through the vagus, splanchnic, mesenteric and pelvic spinal nerves to innervate the abdominal viscera. Figure 1 provides a simplified overview of the anatomy of the mammalian gut-brain axis and its major components. While the general organization of the gut-brain axis appears relatively simple compared to that of the CNS, the neurochemical, anatomical and functional relationships between different populations of gut-brain axis neurons can be highly complex (Anlauf et al., 2003; Travagli et al., 2003; Powley et al., 2005; Lomax et al., 2006; Bertrand, 2009; Brierley, 2010; Fox, 2013). Notably, the anatomical study of gut-brain axis has a remarkably long history. For instance, the vagus nerve was already known to Galen (circa A.D. 130–200) (Ackerknecht, 1974). Furthermore, Renaissance physicians were aware of the importance of the nerve supply to the gut in discerning, as put by Ambroise Paré (Paré, 1968), what is troublesome to the bowels. However, the detailed anatomy of the gut-brain axis remained inaccessible to biologists for a long time because the nerves immediately cease to be distinguishable as they penetrate into peripheral organs. Thus, it was not until the late nineteenth century that postganglionic neurons located in the gastrointestinal wall (also known as enteric neurons) were discovered by Auerbach and Meissner (Meissner, 1857; Auerbach, 1863). Enteric neurons, along with postganglionic neurons in the gallbladder and pancreas, are part of the gut-brain axis, as they receive direct input from, and transmit information to, the rest of the autonomic nervous system; however, enteric neurons are also capable of operating independently of the gut-brain axis (Gershon, 1981; Morris et al., 1985; Mawe et al., 1997; Powley, 2000).

Using Golgi staining (DeFelipe, 2010), histologists in the beginning of the twentieth century continuously refined our understanding of the intricate networks of neurons of the gut-brain axis. Despite its indubitable value, Golgi staining remained capricious. As a result, neuroanatomists have developed more reliable ways of interrogating the organization of the gut-brain axis in recent decades. First, a large number of anatomical studies using the injection of retrograde tracers or neurotropic viruses into visceral tissues have revealed the connections between autonomic nerves and their gastrointestinal targets (Sharkey et al., 1984; Sterner et al., 1985; Altschuler et al., 1989; Rinaman et al., 1999; Rinaman and Schwartz, 2004). Although useful, these newer approaches did not provide a means of labeling autonomic peripheral endings. In addition, retrograde tracing techniques (gut-to-brain) using neurotropic viruses or tracers to verify connections between autonomic neurons and peripheral tissues can potentially produce false-positive results (Fox and Powley, 1986; Berthoud et al., 2006). Moreover, the interpretation of data obtained with neurotropic rabies virus (which travels in a multisynaptic manner) is complicated by the fact that the vagus nerve innervates sympathetic and pelvic ganglia (Berthoud and Powley, 1993), which makes it difficult to ascertain the neural routes taken by these viruses. Alternatively, immunolabeling can be useful in identifying select neuropeptides, neurotransmitters, enzymes and receptors found in vagal and/or spinal visceral endings (Furness and Costa, 1980; Green and Dockray, 1987; Patterson et al., 2002; Chiocchetti et al., 2003; Wang and Neuhuber, 2003; Lindsay et al., 2006; Phillips and Powley, 2007; Mitsui, 2009; Bellier and Kimura, 2011). Unfortunately, specific molecular markers that could be used to selectively label different types of autonomic nerve endings in a systematic manner are still lacking. In addition, the staining obtained with antibodies might not always be distributed evenly in a neuron, and the expression levels of many molecular markers can fluctuate due to experimental conditions. Second, studies employing anterograde tracer (brain-to-gut) injections have been essential to our current understanding of the basic morphology and anatomical distribution of gut-brain axis neurons (Berthoud et al., 1991; Fox et al., 2000; Wang and Powley, 2000; Walter et al., 2009; Zagorodnyuk et al., 2010). Needless to say, those previous studies have served as invaluable references for scientists interested in the gut-brain axis and have revealed many different types of specialized neuronal endings with distinct shapes, functions and tissue distributions in the gut. Details of the variety of receptors found in the gut are available in several publications (Fox et al., 2000; Berthoud et al., 2004; Powley et al., 2011). On a more practical level, however, tracer experiments inherently produce variable results and some degree of tissue damage at the site of injection. Furthermore, tracer injections are laborious and are not always compatible with long-term physiological experiments given the relatively short life of conventional neural tracers, and, in the case of peripheral ganglia, tracer injections remain technically challenging.

Due to the aforementioned limitations, there are still gaps in our knowledge of the impact of various physiological and pathophysiological conditions on gut innervation. A better understanding of the anatomy and plasticity of the gut-brain axis will help to advance our understanding of autonomic neural circuits and numerous chronic diseases that affect the gut-brain axis, including but not limited to inflammatory bowel diseases, metabolic syndrome, visceral pain, and eating disorders (Mayer and Collins, 2002; Powley et al., 2005; Faris et al., 2008; Blackshaw et al., 2010; Cluny et al., 2012; Larauche et al., 2012; Raybould, 2012). The primary goal of this article is to review the principles, advantages, and limitations of various approaches that permit the genetic labeling of specific populations of neurons within the gut-brain axis in temporally and spatially controlled manners. We will focus on components of the vagus and spinal nerves that innervate the gut; however, the methods described in this article can also be applied to other components of the peripheral nervous system (PNS). Further information on the mapping of peripheral nociceptive, somatosensory and somatomotor pathways using reporter mice can be found elsewhere (Tucker et al., 2001; Nguyen et al., 2002; Basbaum and Braz, 2010; Li et al., 2011; Whitney et al., 2011). Although this article focuses on the anatomy of the adult gut-brain axis (rather than its physiology), the usefulness of transgenic tools in studying the metabolic functions of the gut-brain axis will be briefly discussed in the last section.

## Labeling gut-brain axis neurons with transgenic technologies

### Surrogate reporters

#### Principle, advantages, and limitations

Several mouse models that express fluorescent reporter proteins in subsets of gut-brain axis neurons have been described in the literature (Table 1). These mice generally possess a transgene incorporating the regulatory elements of a gene endogenously expressed in gut-brain axis neurons and a green fluorescent protein (GFP) gene or one of its variants. Different approaches for inserting a transgene into the genome exist, but most of the mice discussed in this article were generated either using bacterial artificial chromosome (BAC transgene) or knock-in (inserted in the endogenous allele) approaches. Both approaches have advantages and issues, which are described in more specialized articles (Gong et al., 2003; Dhaliwal and Lagace, 2011; Heffner et al., 2012; Murray et al., 2012). Nonetheless, it is important to remember that the insertion of a transgene into the genome with the BAC approach is random. Due to this random insertion, transgene expression does not always perfectly recapitulate that of the endogenous gene. In contrast, the knock-in approach produces reporter expression that is under the control of endogenous regulatory sequences, which results faithful expression patterns. In all cases, ectopic transgene expression can occur, and the distribution of the reporter must be systematically compared to that of the endogenous gene of interest.

**Table 1:** **Non-exhaustive list of the genetic models employed to introduce reporter expression in the rodent gut-brain axis**.

**Mouse line or virus name**	**Construct**	**Reporter(s)**	**Labeled gut-brain axis neurons**	**Reference(s)**
**CONSTITUTIVE EXPRESSION OF REPORTER**				
CGRPα-GFP	Knockin of eGFPf in *Calca* locus	eGFPf	CGRPα DRG neurons	McCoy et al., 2012
ChAT^BAC^-eGFP	BAC with 78 and 36 kb at 5′and 3′ ends of *Chat* and eGFP in exon2	eGFP	Cholinergic neurons	Tallini et al., 2006
ChAT-6417-LacZ	*LacZ* driven by 5′ fragments of the mouse *ChAT* gene	β-gal		Naciff et al., 1999
ChAT-tauGFP	BAC with entire *Chat* gene and tauGFP in exon3	tauGFP		Grybko et al., 2011
DBH-*lacZ*	*LacZ* driven by 5′ fragments of the human *DBH* gene	β-gal	Noradrenergic neurons	Mercer et al., 1991
GAD-EGFP	eGFP driven by 1.2 kb of regulatory elements upstream of mouse *Gad1*	eGFP	Gabaergic NTS neurons	Oliva et al., 2000; Bailey et al., 2008; Gao et al., 2009
GHSR-eGFP	BAC containing eGFP driven by *GHSR* promoter	eGFP	Subgroup of preganglionic sympathetic neurons	Furness et al., 2011, 2012
MC4R-GFP	BAC containing Tau-sGFP inserted in the ATG site of *Mc4r*	tau-sGFP	Subgroup of NG, DMV and DRG neurons	Kishi et al., 2003; Gautron et al., 2010a, 2012
Peripherin-eGFP	BAC with eGFP driven by the entire human h*PRPH-1* gene	eGFP	Peripherin-containing neurons	McLenachan et al., 2008
*Phox2b*-H2BCFP	BAC with Histone2B-fused with cerulean inserted into the first exon of *Phox2b* locus	cerulean	Autonomic neural crest-derived cells	Corpening et al., 2008
POMC-EGFP	EGFP inserted in exon 2 of murine *Pomc* with 13 kb of 5′ and 2 kb of 3′ regulatory sequences	eGFP	NTS POMC neurons	Fan et al., 2004; Appleyard et al., 2005; Padilla et al., 2012
**CRE-DEPENDENT EXPRESSION OF REPORTER**				
ChAT-IRES-Cre	IRES-Cre inserted in *ChAT* exon 15	GFP, tdTomato	Cholinergic neurons	Rossi et al., 2011; Gautron et al., 2013a
ChAT-Cre	BAC with Cre inserted into the first exon of *ChAT* locus	eGFP	Cholinergic neurons	Gong et al., 2003
Na_v_1.8-Cre	Cre knocked into *Na_v_1.8* locus	β-gal, tdTomato	Na_v_1.8-expressing afferents	Stirling et al., 2005; Gautron et al., 2011; Shields et al., 2012
Na_v_1.8-Cre (or SNS-Cre)	BAC of *Na_v_1.8* locus containing Cre	β-gal		Agarwal et al., 2004
Phox2b^cre^	BAC containing Cre inserted in *Phox2b* exon 2	YFP	All autonomic neurons but sympathetic preganglionic	D'Autreaux et al., 2011
Phox2b-Cre	BAC containing Cre inserted in *Phox2b* exon 1	EGFP	DMV and NTS	Gong et al., 2003
Phox2b-Cre	Cre driven by *Phox2b* with >75 kb at 3′ and 5′ ends	GFP, β-gal, tdTomato	DMV, NG, subset of NTS and enteric neurons	Rossi et al., 2011; Scott et al., 2011; Gautron et al., 2013b
POMC-Cre	BAC containing *Pomc* locus with Cre inserted in exon 2	eGFP, tdTomato	NTS POMC neurons	Balthasar et al., 2004; Padilla et al., 2012
TH-Cre	IRES-Cre knocked in 3′UTR of *TH* gene	YFP, β-gal	Catecholaminergic neurons	Lindeberg et al., 2004; Obermayr et al., 2013
**VIRALLY DELIVERED REPORTER**				
AAV (serotypes)	CMV-driven eGFP	eGFP	Majority of NG neurons	Kollarik et al., 2010
AdRSV*lacZ*	RSV-driven *LacZ*	β-gal	Majority of cultured NG neurons	Meyrelles et al., 1997
AdCMV*lacZ*	CMV-driven *LacZ*			
AdRSV*gfp*	RSV-driven GFP	GFP		
PRSx8-AlstR-eGFP-LV	eGFP driven by Phox2b-activated promoter	eGFP	DMV neurons at site of injection	Mastitskaya et al., 2012
PRSx8-ChIEFtdTomato-AV	tdTomato driven by Phox2b-activated promoter	tdTomato		
rAAV8-GFP	CMV-driven GFP	GFP	Subgroup of DRG neurons innervating the colon	Storek et al., 2008; Vulchanova et al., 2010; Schuster et al., 2013

For abbreviations and references, see the text.

GFP is a widely used fluorescent reporter that is well-transported and allows for the labeling of terminal endings in both the periphery and CNS. In addition, constitutive reporters closely reflect the endogenous expression of one particular gene of interest at any given time and can allow for the labeling of cells that are otherwise difficult to detect using conventional anatomical methods (Padilla et al., 2012). GFP reporters, however, are not without caveats. For instance, GFP is not always bright enough to be observed without immunostaining (Liu et al., 2003). Fortunately, antisera against GFP that can be used to stain GFP-containing structures are widely available. The marking of cells with GFP reporters is not permanent, and, consequently, these reporters can be useful in studies that aim to examine dynamically regulated proteins (via the resulting up- or down-regulation of GFP). However, this feature can also be a drawback if the primary goal of the study is fate mapping or the permanent labeling of a group of neurons across physiological circumstances. Finally, GFP has been reported to be potentially toxic to brain cells at high expression levels (Howard et al., 2008). Although it does not have the versatility of fluorescent reporters, another reporter protein commonly employed in molecular biology is the β-galactosidase enzyme, which is encoded by the *lacZ* gene. Cells expressing *lacZ* can be labeled in straightforward manner with a blue dye.

#### Review of available tools

The choline acetyltransferase (*ChAT*) gene encodes the enzyme necessary for the synthesis of acetylcholine, the principal neurotransmitter of preganglionic parasympathetic and sympathetic neurons and a large proportion of enteric neurons (Arvidsson et al., 1997). A transgenic mouse with *lacZ* expression that is restricted to cholinergic neurons was generated more than a decade ago (Naciff et al., 1999). This study also established the regulatory sequence in the *ChAT* locus that is necessary to drive specific *lacZ* expression in cholinergic neurons. Dorsal motor nucleus of the vagus (DMV) neurons have been stained in this mouse, but other gut-brain axis neurons have not been examined. More recently, ChAT^BAC^-eGFP and ChAT^BAC^-tau-GFP animals were generated using a BAC strategy (Tallini et al., 2006; Grybko et al., 2011). In both lines, the expression of either enhanced GFP (eGFP) or tau-GFP is under the control of the *ChAT* promoter. Tau is a microtubule-binding protein and, consequently, tau-GFP fusion protein is more effectively transported to terminal processes than GFP alone. As anticipated, the fluorescence in these two lines has been reported in all cholinergic neurons including, DMV and enteric neurons. A study by Grybko and colleagues demonstrated bright native GFP fluorescence that perfectly colocalized with ChAT immunoreactivity. Although these animals have not been characterized in depth (e.g., with ectopic expression), they appear to be useful for the study of cholinergic gut-brain axis neurons.

**Figure 2:**
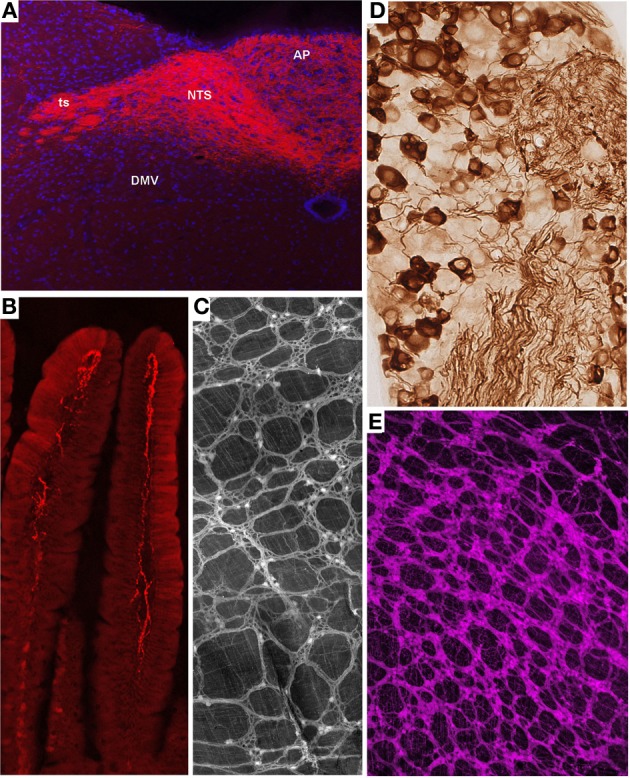
**Representative examples of transgenic approaches that enable the marking of restricted gut-brain axis neurons in the mouse. (A)** Vagal sensory neurons in the NG of MC4R-GFP mouse stained with an anti-GFP antibody and diaminobenzidine. Notably, both the cell bodies and axons of vagal sensory neurons are clearly labeled. **(B,D)** Fluorescently labeled terminal fibers in the dorsovagal-complex and duodenum mucosa of a Na_v_1.8-Cre-tdTomato mouse. **(C)** Whole-mount of the ileal myenteric plexus of a Phox2b-Cre-tdTomato mouse revealing vagal extrinsic innervation and isolated innervation of enteric neurons. **(E)** Whole-mount of the gastric myenteric plexus of a ChAT-Cre-tdTomato mouse containing fluorescent preganglionic fibers intermingled with cholinergic enteric neurons that reveals the myenteric plexus. The above images are all unpublished and were generated in our laboratory using mouse lines described in the article.

Melanocortin-4 receptor (MC4R) is an important molecular player in the regulation of feeding, metabolic, and autonomic functions (Cone, 2005). Not surprisingly, MC4R is expressed in gut-brain axis neurons including subsets of both motor and sensory vagal and spinal neurons (Kishi et al., 2003; Gautron et al., 2010a, 2012). MC4R-GFP mice that express tau-sapphire-GFP fusion protein under the control of the MC4R regulatory sequences have been generated (Liu et al., 2003) (Figure 2A). These mice express GFP in subgroups of nodose ganglion (NG) and DMV neurons, as well as in preganglionic sympathetic neurons (Liu et al., 2003; Gautron et al., 2010a), and the GFP is present only in MC4R-expressing neurons. Interestingly, GFP is transported to the terminal endings of vagal neurons within the gastrointestinal tract and, hence, reveal the peripheral targets of MC4R in the hepatic artery, myenteric plexus and intestinal mucosa (Gautron et al., 2010a).

A mouse that expresses eGFP under the ghrelin receptor (GHSR) promoter was recently characterized (Furness et al., 2011). Simply expressed, ghrelin is a hormone secreted by the stomach epithelium that promotes hunger and modulates autonomic functions (Nogueiras et al., 2008). Using double-labeling and retrograde tracer experiments, a subgroup of preganglionic sympathetic neurons, including neurons connected to the gut (as evidenced by retrograde tracing experiments), has been identified to contain eGFP (Furness et al., 2012). The projections of these neurons terminating in sympathetic ganglia are nicely labeled by eGFP immunohistochemistry. These data suggest that GHSR-eGFP mice are a valid model for identifying GHSR-expressing gut-brain axis neurons. As GHSR mRNA has been reported to be present in NG and DMV neurons (Date et al., 2002; Zigman et al., 2006), further work to determine whether vagal neurons are labeled in this reporter model is warranted.

GABAergic interneurons located in the nucleus of solitary tract (NTS) play a critical role in vago-vagal reflexes (Bailey et al., 2008). GAD-EGFP mice express eGFP under the control of the glutamic acid decarboxylase-67 gene (aka *Gad1*) (Oliva et al., 2000). The vast majority of GFP-positive cells are immunoreactive for GABAergic markers. Many brain sites known to contain GABAergic neurons do not display GFP fluorescence, which suggests GFP underexpression. Brainstem slices from the GAD-EGFP mouse have been employed to facilitate the patch-clamping of interneurons in the dorsovagal complex to conduct detailed measurements of their electrophysiological properties in response to vagal afferent stimulation (Bailey et al., 2008; Gao et al., 2009).

Paired-like homeobox 2b (Phox2b) is a transcription factor that is critically involved in the early differentiation of autonomic and viscerosensory pathways (Brunet and Pattyn, 2002). The neural precursors of many gut-brain axis neurons express Phox2b during development including virtually all parasympathetic, enteric and sympathetic ganglia neurons (Tiveron et al., 1996). One mouse line expressing a fused Histone2B-cerulean protein under the control of *Phox2b* was generated using a BAC strategy to mark enteric progenitors (Corpening et al., 2008). The reporter strictly localizes to the nucleus, rendering the identification of adjacent neurons straightforward. Expression of this transgene has been demonstrated in Phox2b cells in the CNS, sympathetic chain and enteric system, which agrees with the known distribution of Phox2b. However, it is not clear whether vagal neurons express the transgene. Double-labeling with an antibody against Phox2b further validated the genuine distribution of the transgene in Phox2b cells in the developing and adult gut. Thus, far, this model has primarily been used to monitor the migration of enteric progenitors in the developing murine gut and to identify adult neural crest derived cells (Corpening et al., 2008).

Proopiomelanocortin (POMC) is a precursor of several pituitary and hypothalamic peptides that is important in neuroendocrine and metabolic functions (Liu et al., 1992; Cowley et al., 2001). The NTS contains a small population of POMC-expressing neurons (Padilla et al., 2012). Unlike other POMC cells, NTS POMC neurons produce little POMC transcript, rendering their detection challenging with conventional neuroanatomical tools. Fortunately, a mouse expressing eGFP under the control of the regulatory sequence of the murine *Pomc* gene has been generated and widely used to visualize NTS POMC neurons (Cowley et al., 2001; Fan et al., 2004; Appleyard et al., 2005; Padilla et al., 2012). Specifically, electrophysiological studies using POMC-EGFP animals have identified NTS POMC neurons as part of a vagovagal circuit that is implicated in satiation (Appleyard et al., 2005).

Other GFP reporter lines that are potentially interesting for the study of gut-brain axis neurons include the peripherin-eGFP (McLenachan et al., 2008) and αCGRP-farnesylated-GFP mice (McCoy et al., 2012). Peripherin is an intermediate filament that is not unique to the gut-brain axis but is widely distributed in the PNS (Troy et al., 1990). The peripherin-eGFP mouse shows fluorescence in many peripheral sensory neurons and enteric neurons and their peripheral projections (McLenachan et al., 2008). It remains unclear whether GFP expression recapitulates endogenous peripherin gene expression. Calcitonin gene-related peptide (CGRP)-α and -β are neuropeptides produced in enteric, nociceptive and viscerosensory neurons (Mulderry et al., 1988). The CGRPα-GFP mouse shows GFP immunoreactivity in many dorsal root ganglion (DRG) neurons and nerve bundles in the intestines but not in enteric neurons (McCoy et al., 2012). While all CGRP-immunoreactive DRG neurons display GFP, approximately 30% of GFP neurons are not CGRP immunoreactive, which raises the question of transgene ectopic expression and/or subthreshold expression of CGRP.

The dopamine β-hydroxylase (*DBH*) gene is responsible for the synthesis of noradrenaline in the autonomic nervous system (Elfvin et al., 1993). The human β-hydroxylase gene has previously been employed to drive *LacZ* expression in the mouse to identify noradrenergic neurons (Mercer et al., 1991). Several lines have been produced, including one that expresses β-gal fused with a nuclear translocation signal. As anticipated, transgene expression has been reported in sympathetic ganglia, cranial parasympathetic ganglia, the enteric nervous system, and in a small portion of neurons in the peripheral sensory ganglia (Mercer et al., 1991). However, the presence of transgene expression in cholinergic ganglia may be due to ectopic expression.

Finally, while the development of the enteric nervous system is a field of study that is too vast to be reviewed in the current article, it is noteworthy that a number of transgenic reporters have been created to study the fate mapping and differentiation of enteric neurons during early development (Hanna et al., 2002; Young et al., 2004; Deal et al., 2006; Corpening et al., 2008, 2011; Mundell et al., 2012). To avoid oversimplifying this complex area of research, we deliberately avoid reviewing these models.

### Cre/LoxP technology

#### Principle, advantages, and limitations

**Figure 3:**
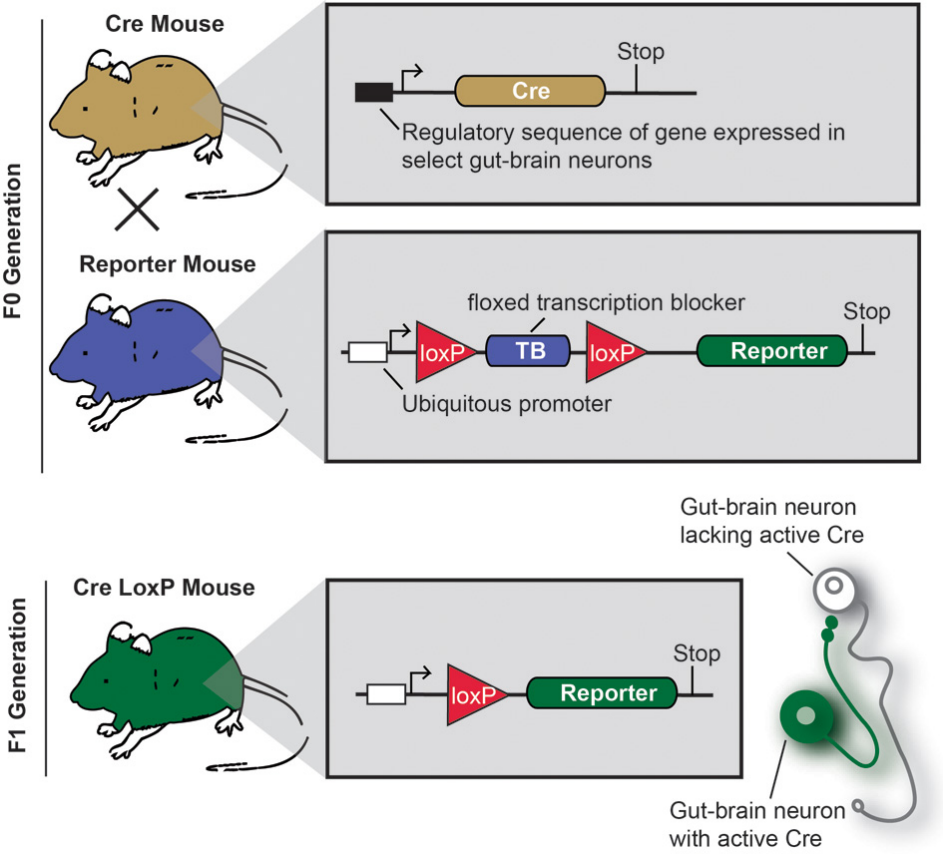
**Principle of an experiment using the Cre-LoxP system to label specific gut-brain axis neurons in the mouse**.

Cre/LoxP technology deserves specific attention, as this approach profoundly changed how neuroanatomy is conceived and performed (Dymecki and Kim, 2007; Livet et al., 2007; Madisen et al., 2010; Weissman et al., 2011). Figure 3 describes the principles of the Cre/LoxP technology applied to the labeling of gut-brain axis neurons. Briefly, by transgenically directing the expression of Cre-recombinase (Cre) to discrete populations of neurons, including gut-brain axis neurons (using BAC or gene targeting), it is possible to induce and modulate the expression of a target fluorescent reporter in a Cre-responsive manner. Recently, different laboratories have taken advantage of available mouse Cre lines to specifically manipulate gene expression in autonomic neurons. To label neurons, select Cre mice can be systematically crossed with reporter mice that possess a loxP-flanked stop cassette that prevents the expression of *LacZ* or a fluorescent reporter protein. In Cre-expressing neurons, however, the transcriptional termination sequence is excised allowing reporter production (Madisen et al., 2010). As an example, mice that express tdTomato in a Cre-dependent manner are often utilized because tdTomato is one of the brightest red fluorescent proteins available (Shaner et al., 2004). Thus, native fluorescence can be observed not only in the cell bodies but also the central relays and peripheral endings, which reveals the full extent of Cre-expressing neuron connectivity. One major advantage of this approach is that it allows for the permanent labeling of non-replicating cells with a reporter protein. A few examples the utilization of the aforementioned strategy of labeling gut-brain axis neurons are described below (see also Table 1). Nonetheless, caution must be taken when interpreting the distribution of any given Cre-expressing cell population. Indeed, Cre expression can occur transiently in developing neurons that do not express the transgene of interest in adulthood, which results in the labeling of cells other than the intended target neurons (Heffner et al., 2012; Padilla et al., 2012). Thus, off-target and inconsistent Cre activity must be considered as potential drawbacks when using the Cre-LoxP approach.

#### Review of available tools

Na_v_1.8 is a tetrodotoxin-resistant sodium voltage-gated channel that is enriched in C-fiber peripheral afferents, including a significant proportion of spinal nociceptors, and subsets of low-threshold mechanoreceptors (Djouhri et al., 2003; Fukuoka et al., 2008; Shields et al., 2012). It has been clearly established that Na_v_1.8 is essential to the electrogenesis of nociceptors and, as a corollary, certain pain phenotypes including visceral pain (Laird et al., 2002; Zimmermann et al., 2007). More surprisingly, expression of Na_v_1.8 has been demonstrated in a majority of vagal sensory neurons (Stirling et al., 2005; Gautron et al., 2012). Transgenic mice expressing Cre under the control of the Na_v_1.8 promoter have been generated and characterized (Stirling et al., 2005). In these mice, the translational start-site of Na_v_1.8 was substituted with that of Cre recombinase. Mice carrying one Na_v_1.8-cre allele show normal pain behavior, and their DRG neurons exhibit normal electrophysiological properties (Stirling et al., 2005). However, mice with two Cre alleles are equivalent to Na_v_1.8 knock-out mice and, thus, tetrodotoxin-sensitive currents are absent in these mice. Embryonic and adult Cre recombinase expression reported by β-galactosidase activity is specific to small diameter neurons in the DRG and trigeminal ganglion and many neurons in the NG. Other than a few neurons in the superior cervical ganglion, no Cre activity has been observed in peripheral tissues or the CNS. We, and others, crossed Na_v_1.8-Cre mice with tdTomato reporter animals (Gautron et al., 2011; Shields et al., 2012). In the offspring, all Na_v_1.8-expressing neurons, including many vagal and spinal afferents and their connections within the gut and CNS, are fluorescently marked (Gautron et al., 2011). As with other examples, we were able to visualize vagal tension and mucosal endings in the myenteric plexuses of Na_v_1.8-Cre-tdTomato mice (Figures 2B,D). Importantly, the numbers and distribution patterns of tdTomato-positive cells in the DRG and NG are directly comparable to those of Na_v_1.8 mRNA, suggesting that Cre activity occurs only in Na_v_1.8-expressing neurons. Another mouse expressing Cre under the Na_v_1.8 promoter also exists (Agarwal et al., 2004). Because this model was generated with a BAC, it has the advantage of not disrupting the endogenous *Na_v_ 1.8* gene in contrast to the knock-in approach described previously.

ChAT-Cre mice have recently been generated by targeting the *ChAT* gene with IRES-Cre (Rossi et al., 2011). Double-labeling experiments have shown that these mice faithfully express Cre in cholinergic neurons both in the CNS and PNS. When crossed with a tdTomato reporter line, the offspring produce tdTomato in all cholinergic neurons, which allows for distinct labeling of the cell bodies and projections of all preganglionic sympathetic and parasympathetic neurons and cholinergic enteric neurons (Gautron et al., 2013a) (Figure 2C). Importantly, Cre activity appears to be limited to ChAT-immunoreactive cells. Compared to the ChAT-GFP lines described previously, ChAT-Cre mice offer the advantage of allowing the reporter to be invariably expressed in ChAT cells regardless of ChAT expression levels. This is important because neuronal ChAT mRNA levels may be differentially regulated under different physiological circumstances (Gibbs, 1996; Castell et al., 2002). Several mouse lines expressing Cre under the control of *ChAT* have also been created in the context of the Gensat project (Gong et al., 2003). These mice appear to show restricted Cre activity in cholinergic neurons including DMV neurons. Little information is available about the distribution of Cre in other gut-brain axis neurons.

A mouse expressing Cre under the control of *Pomc* regulatory elements has been generated with a BAC (Balthasar et al., 2004). POMC-Cre mice crossed with different inducible reporters have been used in several laboratories to identify POMC hypothalamic and/or NTS neurons (Balthasar et al., 2004; Huo et al., 2006; Zheng et al., 2010). A recent study revealed significant discrepancies between cells with POMC-Cre activity and POMC-EGFP transgene expression (Padilla et al., 2012). Specifically, the populations of NTS cells labeled by the coexpression of both the POMC-EGFP and POMC-Cre overlap minimally, which raises again the issue of possible ectopic and off-target expression.

Several Phox2b-Cre mice have recently been generated by different laboratories (D'Autreaux et al., 2011; Rossi et al., 2011). Phox2b-Cre mice from the group of Dr. Brunet were crossed with a YFP reporter mouse to label Phox2b-Cre-expressing neurons (D'Autreaux et al., 2011). As a result, Phox2b^CreYFP^ embryos show YFP staining in the progenitors of many neurons of the AP, NTS, DMV, NG, and the enteric nervous system. Phox2b-Cre mice created by the Gensat project display Cre activity in the NTS and DMV (Gong et al., 2003). The extent to which this mouse exhibits Cre in peripheral ganglia is unknown. The Phox2b-Cre line generated by the Elmquist group was crossed with a GFP or *lacZ* reporter mouse and proven to express Cre in all NG, DMV, and a subgroup of NTS neurons (Rossi et al., 2011; Scott et al., 2011). The latter mouse did not display Cre activity in sympathetic neurons and displayed Cre activity in only a small subset of enteric neurons, suggesting that, presumably due to positional effects, the expression of Cre underestimates that of the endogenous *Phox2b* gene. Nonetheless, this mouse has proven useful for selectively tagging the vagus nerve. In Phox2b-Cre-tdTomato mice, distinguishing vagal motor vs. sensory endings remains difficult in locations in which they are intermingled (i.e., the myenteric plexus); however, the anatomical integrity of the entire vagus nerve can easily be gauged (Gautron et al., 2013b) (Figure 2E). In the latter study, we also showed that Roux-en-Y gastric bypass surgery results in significant vagal denervation of the stomachs of Phox2b-Cre-tdTomato mice. This finding is in agreement with observations that suggest significant morphological and electrophysiological changes in vagal neurons following gastrointestinal surgical intervention in the rat (Phillips and Powley, 2005; Guijarro et al., 2007; Browning et al., 2013b).

Postganglionic sympathetic neurons express tyrosine hydroxylase (TH), the rate-limiting enzyme in catecholamine biosynthesis (Elfvin et al., 1993). Several mice expressing Cre under the control of the TH promoter have been generated in the past (Gelman et al., 2003; Lindeberg et al., 2004; Savitt et al., 2005). Using a TH-Cre knock-in, catecholaminergic gut-brain axis neurons, including sympathetic postganglionic neurons and a small population of enteric neurons transiently expressing TH during development, can be marked (Lindeberg et al., 2004; Obermayr et al., 2013). The patterns of Cre activity have been validated using TH staining, Cre mRNA *in situ* hybridization and reporter distributions (β-gal and YFP).

Remarkably, numerous reagents expressing Cre under the control of transcription factors implicated in the development of neural–crest derived tissues (other than Phox2b) are available (Druckenbrod and Epstein, 2005; Stine et al., 2009; Mundell et al., 2012). While these models are indispensable for tracking enteric progenitors migrating in the developing gut, as mentioned before, it is beyond the scope of this article to review the transgenic tools used in developmental biology. Lastly, new Cre lines are continuously being generated and are readily available to investigators. A few examples include neuropeptide Y-, Transient receptor potential vanilloid receptor 1-, TH-, advilin- and peripherin-Cre mice, which have all been demonstrated to induce Cre expression in populations of peripheral afferents and/or brain neurons (Zhou et al., 2002; Gelman et al., 2003; Braz and Basbaum, 2009; Mishra et al., 2011; Zurborg et al., 2011). While the genes employed to drive Cre expression in the latter transgenics are known to be transcriptionally active in gut-brain axis neurons, insufficient information is available to assess their usefulness in targeting the gut-brain axis. Therefore, we will not include these animals in our review.

### Virally mediated gene delivery

#### Principles, advantages, and limitations

Stereotaxic injections of viral vectors of varying packaging capacity, capsid serotype, and cellular tropism are commonly used to deliver transgenes that encode fluorescent proteins into the CNS (Klein et al., 2002; Tenenbaum et al., 2004; Luo et al., 2008; Betley and Sternson, 2011). As illustrated by the examples below, viral vectors appear adequate to transfer GFP expression in gut-brain axis neurons; however, few studies have attempted to do this (Table 1). Generally speaking, viral vectors confer long-term gene expression and are deliverable to laboratory rodents, non-human primates and to humans for therapeutic purposes (Kay et al., 2000; Christine et al., 2009; Bu et al., 2012). While immune responses to viral particles and toxicity are a concern with certain viral vectors (Sawada et al., 2010), recombinant adeno-associated viruses (rAAV) produce little (if any) inflammation (Chamberlin and Saper, 1998; Lowenstein and Castro, 2002), and virally delivered transgenes do not integrate into the host genome (Schnepp et al., 2003). To further minimize neuronal injury, using a glass micropipette coupled to an iontophoretic or air-pressure set-up for injection is preferable (Chamberlin et al., 1998; Krenzer et al., 2011). Interestingly, transgenes transferred by viral vectors can be Cre-dependent, which allows specific gene expression that is limited to molecularly defined neurons at the site of injection (Lazarus et al., 2007; Gautron et al., 2010b; Harris et al., 2012). However, this strategy is inherently limited by the fact that injecting the NG, DRG, or enteric system is technically challenging. Finally, the number of transfected neurons at the site of injection is always inherently variable, and it is not completely clear whether all gut-brain axis neurons are equally sensitive to viral transfection.

#### Review of available tools

Mastitskaya and colleagues recently demonstrated the feasibility of delivering a few different transgenes encoding engineered receptors (i.e., allatostatin and channel rhodopsin receptors) into the rat DMV using a lentivirus and an adenovirus (Mastitskaya et al., 2012). Of note, both vectors incorporated an artificial Phox2 promoter to ensure the preferential expression of the transgenes in the DMV. Each transgene was also coupled with the expression of a specific fluorescent reporter, either eGFP or tdTomato. As a result, DMV neurons in brainstem slices could be visualized by their respective eGFP and tdTomato fluorescence and then patched for electrophysiological recordings. This study is a good illustration of the usefulness of viral vectors in manipulating gene expression in DMV neurons. There are few studies that have attempted virally mediated gene delivery in neurons residing in the peripheral ganglia. Intrathecal delivery of rAAVs carrying a GFP transgene has been performed in rats and mice and resulted in the labeling of DRG neurons (Storek et al., 2008; Vulchanova et al., 2010). Despite the variable transfection efficiency of this approach, it was recently reported that this approach can be used to label subsets of DRG neurons that supply the mouse colon (Schuster et al., 2013). Specifically, GFP-positive spinal sensory terminals have been observed in the colonic myenteric plexus and mucosa of injected mice. Thus, far, only one study has directly administered AAVs of different serotypes into the guinea pig NG (Kollarik et al., 2010). Stable expression of eGFP was successfully obtained in 50–80% of vagal sensory neurons depending on the serotype. The same study indicated that the injection of an AAV into vagally innervated tissue is sufficient to transfect vagal neurons innervating that tissue (i.e., the esophagus) and that the eGFP-filled terminal endings of transfected vagal sensory neurons can be observed in the periphery including the trachea and esophagus. In the case of vagal sensory neurons, it is also known that a vast majority of primary-cultured dissociated NG neurons can be successfully transfected with adenoviruses driving either *LacZ* or GFP expression (Meyrelles et al., 1997).

## Areas of improvement

Although the rapidly evolving transgenic technologies described in this article are not meant to replace more conventional approaches, they offer numerous advantages over classical tracing and immunohistochemical techniques. In the future, we foresee that the approaches described in this article will change many areas of research that require the visualization of visceral afferents, vagal and enteric neurons, especially for scientists who are not familiar with neuroanatomical techniques. However, transgenic technologies have numerous caveats. First, the paucity of well-characterized mouse lines that permit targeting gut-brain neurons greatly limits our ability to manipulate the full range of the different types of gut-brain axis neurons described in the literature. For example, the development of Cre lines that are adequate to differentiate vagal stretch (intramuscular arrays) vs. tension receptors (intraganglionic laminar endings) from mucosal endings would be useful in clarifying the physiological role of each of the aforementioned vagal endings. It is particularly important considering that subpopulations of vagal afferents may have completely different roles in appetite regulation. Moreover, the ability to differentiate the non-neuronal cells implicated in the normal functioning of the gut-brain axis, such as glial cells and interstitial cells of Cajal, would be useful (McDougal et al., 2011; Powley and Phillips, 2011; Gulbransen and Sharkey, 2012). We expect that this problem will become smaller in the future as the number of strains generated by large-scale Cre-driver projects continues to grow and become available in public repositories (Gong et al., 2003; Murray et al., 2012). The lack of temporal specificity of the models described in this article is another problem. For example, it would be useful to have the ability to restrict Cre to a desired life stage. Among other strategies, this could be achieved using mice that express a Cre-estrogen receptor-fused protein. In these animals, Cre-induced recombination occurs only following the exogenous administration of tamoxifen (Badea et al., 2003), which restricts Cre activity to a desired temporal stage and facilitates cell lineage studies. Likewise, the tetracycline system is a popular technique that is employed to control gene transcription in a reversible and temporally restricted manner (Schonig et al., 2013). More inducible Cre drivers relevant to the gut-brain axis will become available in the future. As mentioned before, ectopic and/or inconsistent transgene expressions are issues that need to be addressed when working with transgenic technologies. Lastly, it must be noted that the physiology of the gut-brain axis has been extensively studied in guinea pigs and rats instead of mice, and these species have not been traditionally targeted for transgenics. However, transgenic rats, including rat Cre-lines, are becoming more widely available (Witten et al., 2011; Schonig et al., 2012).

## Perspectives in metabolic research

Many investigators acknowledge that identifying the mechanisms and pathways underlying the central integration of visceral information is a true challenge that could provide a better understanding of diseases including obesity (Powley et al., 2005; Berthoud, 2008; Yi and Tschop, 2012). The lack of adequate methods to interrogate neural pathways linking the gut and the brain has largely contributed to the slow progress in this area—especially compared to other areas of sensory biology and somatomotor systems. To aid the investigation of these pathways, we and others have begun using and developing new transgenic tools to manipulate gene expression in the gut-brain axis. In the last section, we will briefly review what is known about the implications of gut-brain axis neurons in diabetes and obesity and will make the case that transgenic models can be instrumental in deciphering the role played by the gut-brain axis in chronic metabolic diseases.

Although hepatic glucose flux is primarily under the direct control of insulin (Cardin et al., 2002), numerous studies have found that stimulating vagal efferents alters peripheral glucose flux and insulin secretion (Shimazu and Fujimoto, 1971; Shimazu, 1971; Rohner-Jeanrenaud et al., 1983; Berthoud et al., 1990; Rozman and Bunc, 2004; Peitl et al., 2005). Moreover, intact vagal fibers and capsaicin-sensitive afferents are required for the normal regulation of hepatic and pancreatic functions (Obici et al., 2001; Pocai et al., 2005; Razavi et al., 2006; Uno et al., 2006; Gram et al., 2007) and the anti-diabetic effects of gastric bypass (Troy et al., 2008). Together, these observations strongly support the idea that gut-brain axis neurons significantly contribute to regulating glucose homeostasis. Furthermore, diabetes is a leading cause of injury to the PNS (Westerman et al., 1989; Drel et al., 2006), and these injuries may profoundly alter the functioning of gut-brain axis as suggested by the many gastrointestinal and autonomic symptoms encountered by people with diabetes. Manipulating the neurons that supply the viscera has also been proposed as a potentially relevant weight-loss strategy on the basis of the known regulatory effect of vagal afferents on satiation (Powley et al., 2005). Subdiaphragmatic vagotomy disrupts food consumption in rodents (Phillips and Powley, 1998; Powley et al., 2005) and induces weight-loss in humans (Kral, 1978), but the results of this procedure are difficult to interpret because vagotomy also impairs gastrointestinal motility. In lean animals, most studies agree that the selective surgical, genetic, or capsaicin-induced deafferentation of the vagus nerve results in altered meal patterns (Chavez et al., 1997; Schwartz et al., 1999; Fox et al., 2001; Chi et al., 2004) without causing frank obesity or long-term hyperphagia due to compensatory changes in feeding behavior. In obese animals, however, capsaicin-induced deafferentation of the truncal vagus nerve partially prevents diet-induced obesity (Stearns et al., 2012). Lastly, device–assisted stimulations of the vagus nerve and stomach wall have entered preclinical trials for the treatment of obesity (Aronne and Waitman, 2004; Bodenlos et al., 2007; Camilleri et al., 2008; Val-Laillet et al., 2010), and several laboratories are currently examining the contributions of the different branches of the vagus nerve to the beneficial effects of bariatric surgeries (Bueter et al., 2010; Breen et al., 2012; Shin et al., 2012).

While the above observations taken together link the gut-brain axis to diabetes and obesity, we do not yet possess a clear picture of the contributions of the visceral nerves to the pathophysiologies of chronic metabolic diseases because vagotomy studies have generated confounding results and remain inherently limited in terms of manipulating specific population of vagal afferents. As the vagus nerve is a mixed nerve, surgical techniques, in addition to being technically challenging, also result in full or partial loss of efferent (motor) function. Pharmacological techniques, including the administration of capsaicin to destroy small-diameter sensory neurons, have been used. However, this method also kills some neurons in the CNS without killing all vagal sensory neurons (Ritter and Dinh, 1992; Czaja et al., 2008; Browning et al., 2013a). Additionally, by 60-days post-capsaicin treatment, neuronal nuclei in the NG of rats are not different from controls (Czaja et al., 2008), which limits the applicability of this approach for studying long-term regulation by the sensory vagus nerve. Furthermore, regeneration/plasticity of some sensory terminals after vagotomy has been reported (Phillips et al., 2000). Newly developed genetic tools have emerged to circumvent many of the aforementioned problems. First, transgenic reagents can be particularly useful in studies seeking to knock-out or “reactivate” genes in autonomic pathways relevant to the regulation of peripheral glucose flux and insulin secretion. For example, a few recent studies have employed Cre-LoxP technology to selectively reactivate MC4R expression in preganglionic vagal and sympathetic neurons and demonstrated the key role of this receptor in ameliorating diabetes in MC4R null mice (Rossi et al., 2011; Zechner et al., 2012). Other studies have focused on examining deficits in neurotrophic factor innervation of the gastrointestinal tracts of knockout mice and the physiological consequences of these deficits on feeding (Rossi et al., 2003; Fox, 2013). Second, new neuromodulation techniques have recently been developed that allow the genetic targeting of specific neurons with engineered transmembrane proteins that can exert varied effects on neuronal activity (Aponte et al., 2011; Krashes et al., 2011). Specifically, optogenetic techniques involve light-sensitive proteins known as opsins that alter neuronal activity in response to a blue light. Pharmacogenetic techniques involve G-coupled proteins receptors that only respond to ligands with no other biological activity. While these approaches vary in their kinetics and invasiveness, they both allow the reversible and “remote” control of depolarization or hyperpolarization of targeted neurons within intact neural circuits. To the best of our knowledge, only one study has employed these new strategies to modulate the activity of DMV neurons (Mastitskaya et al., 2012). While this study aimed to interrogate the cardiovascular functions of DMV neurons rather than gut-related functions, it did provide a proof-of-concept experiment demonstrating that opto- and pharmacogenetics have the potential to deeply transform the study of the gut-brain axis. Finally, Cre-LoxP can be used to ablate selective neurons in the CNS or PNS using an inducible diphtheria toxin system (Luquet et al., 2005; Abrahamsen et al., 2008). In the future, these methods could be applied to the gut-brain axis to perform “molecular vagotomies” of specific population of vagal neurons.

The above considerations are meant only to illustrate how transgenic tools can be useful to further our understanding and to predict the beneficial and deleterious consequences of gut-brain axis activity modulation by surgical, pharmacological, or device-assisted means. As the number of transgenic reagents available to gut-brain axis scientists continues to grow and become more sophisticated, we predictable that these new tools will play a critical role in the advancement of our knowledge of the development, morphological plasticity, molecular phenotyping, and connectivity of the healthy and diseased gut-brain axis. In addition to performing neuroanatomy, one can easily imagine many applications for these transgenic tools, including, but not limited to, patch clamp (Bailey et al., 2008; Gao et al., 2009) and flow cytometry sorting (Buehler et al., 2012) of fluorescently tagged neurons. Obviously, research on gut-brain axis neurons is not limited to metabolic diseases but is pertinent to numerous questions relevant to visceral pain, gut flora homeostasis, whole-body inflammation and eating disorders, among other examples (Faris et al., 2008; Andersson and Tracey, 2011; Sharkey and Mawe, 2012). In summary, the rapidly evolving techniques described in this article have become indispensable and empowered both anatomists and physiologists with unique tools to understand better the gut-brain axis in the context of intact animals.

### Conflict of interest statement

The authors declare that the research was conducted in the absence of any commercial or financial relationships that could be construed as a potential conflict of interest.

## Abbreviations

AP: area postrema
BAC: bacterial artificial chromosome
β-gal: β–galactosidase
CNS: central nervous system
CGRP: calcitonin gene-related peptide
ChAT: choline acetyltransferase
CMV: cytomegalovirus
Cre: Cre-recombinase
DBH: dopamine β-hydroxylase
DMV: dorsal motor nucleus of the vagus
DRG: dorsal root ganglion
eGFP: enhanced GFP
eGFPf: enhanced GFP farnesylated
GAD: glutamic acid decarboxylase
GHSR: ghrelin receptor
GFP: green fluorescent protein
IML: intermediolateral cell column
IRES: internal ribosome entry site
MC4R: melanocortin-4 receptor
mp: myenteric plexus
NTS: nucleus of solitary tract
NG: nodose ganglion
PNS: peripheral nervous system
Phox2b: paired-like homeobox 2b
POMC: proopiomelanocortin
PRS: noradrenergic-specific Cis element
preSVG: prevertebral sympathetic ganglion
rAAV: recombinant adeno-associated viruses
RSV: rous sarcoma virus
smp: submucosal plexus
TB: transcription blocker
TH: tyrosine hydroxylase
UTR: untranslated region
X: vagus nerve.
